# Supplementary material for: Characterization of Rosa damascena Callus-Derived Exosome-like Vesicles and Their Multifunctional Activities in Skin-Related Cellular Models
Source: Int J Mol Sci. 2026 May 29;27(11):4938. doi: 10.3390/ijms27114938 (PMC13256896; doi:10.3390/ijms27114938)
Supplement: Supplementary file 1 [file ijms-27-04938-s001.zip › Supplementary Data 5. Representative predicted structures of candidate small RNAs in RSC-EXO.pdf]

Supplementary Data 5. Representative predicted structures of candidate small RNAs in RSC-EXO

Two representative candidate small RNA sequences (LYNE01003137_18835 and LYNE01061673_42069) identified through exploratory computational analysis are shown. These candidates exhibited relatively high read abundance and predicted secondary structures compatible with miRNA-like features; however, their biological function and classification remain unvalidated.

Provisional ID	:	LYNE01003137_18835
Score total	:	619.1
Score for star read(s)	:	3.9
Score for read counts	:	612.4
Score for mfe	:	1.1
Score for randfold	:	1.6
Score for cons. seed	:	
Total read count	:	1213
Mature read count	:	1200
Loop read count	:	0
Star read count	:	13

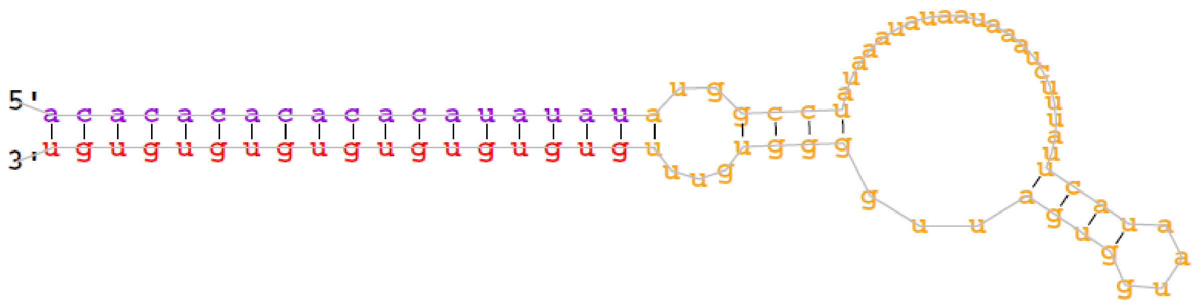

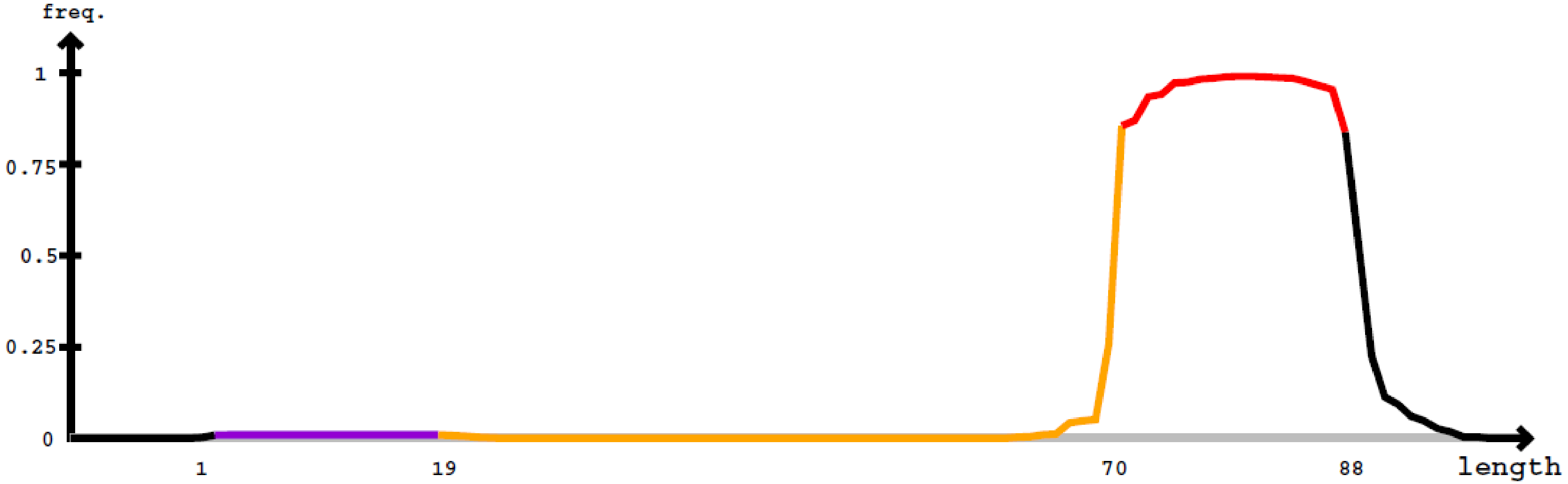

Star Mature

5'- uuucauuuaacacacacacacauauauauggccuauaaauauaauaaaucuuuauucauaauggugauugggguguuugugugugugugugugugugauguguguauuaa -3' obs

uuucauuuaacacacacacacauauauauggccuauaaauauaauaaaucuuuauucauaauggugauugggguguuugugugugugugugugugugauguguguauuaa exp

Provisional ID	:	LYNE01061673_42069
Score total	:	580.8
Score for star read(s)	:	3.9
Score for read counts	:	573.7
Score for mfe	:	1.6
Score for randfold	:	1.6
Score for cons. seed	:	
Total read count	:	1137
Mature read count	:	1123
Loop read count	:	0
Star read count	:	14

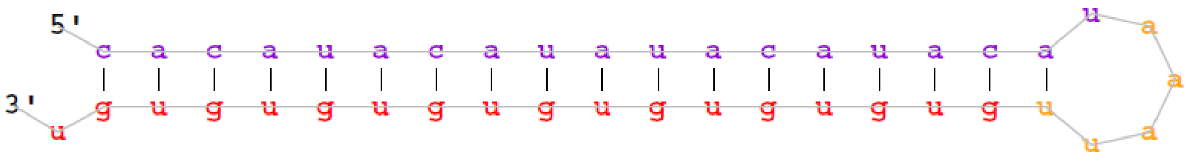

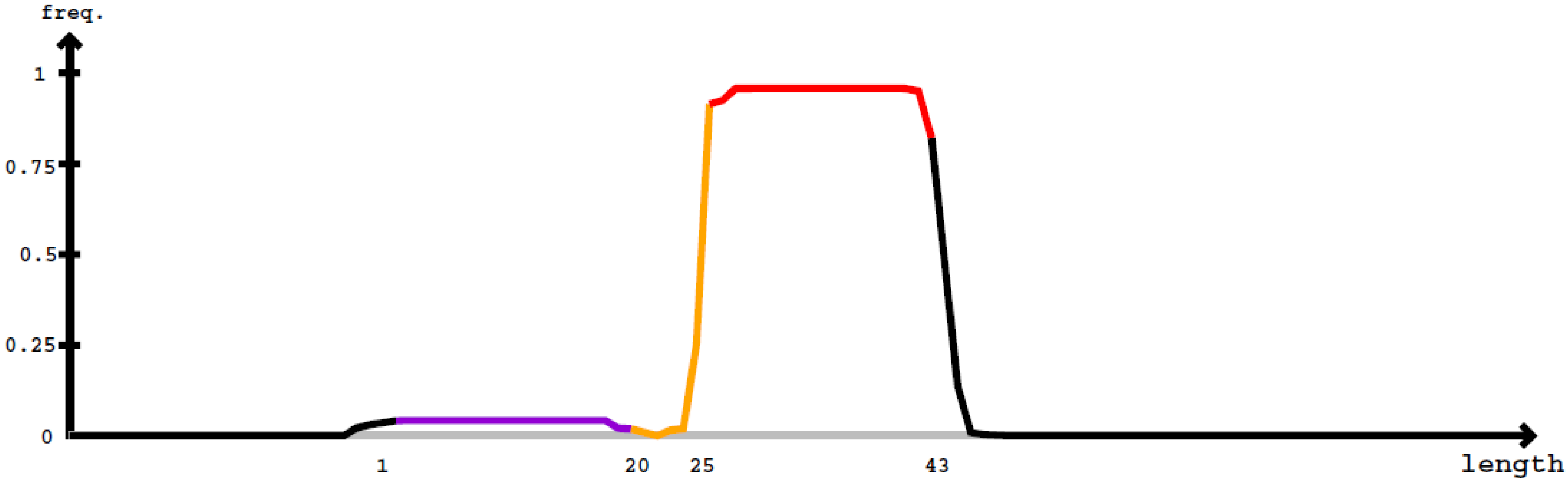

Star Mature

5'- aaaggugaauaacugacuagcuacacauacauauacauacauaaauugugugugugugugugugu auuacauauguauauugacaugcaguuuuucauuuuauuuugau -3' obs

aaaggugaauaacugacuagcuacacauacauauacauacauaaauugugugugugugugugugu auuacauauguauauugacaugcaguuuuucauuuuauuuugau exp
