# Supplementary material for: Characterization of Rosa damascena Callus-Derived Exosome-like Vesicles and Their Multifunctional Activities in Skin-Related Cellular Models
Source: Int J Mol Sci. 2026 May 29;27(11):4938. doi: 10.3390/ijms27114938 (PMC13256896; doi:10.3390/ijms27114938)

## Slide 1
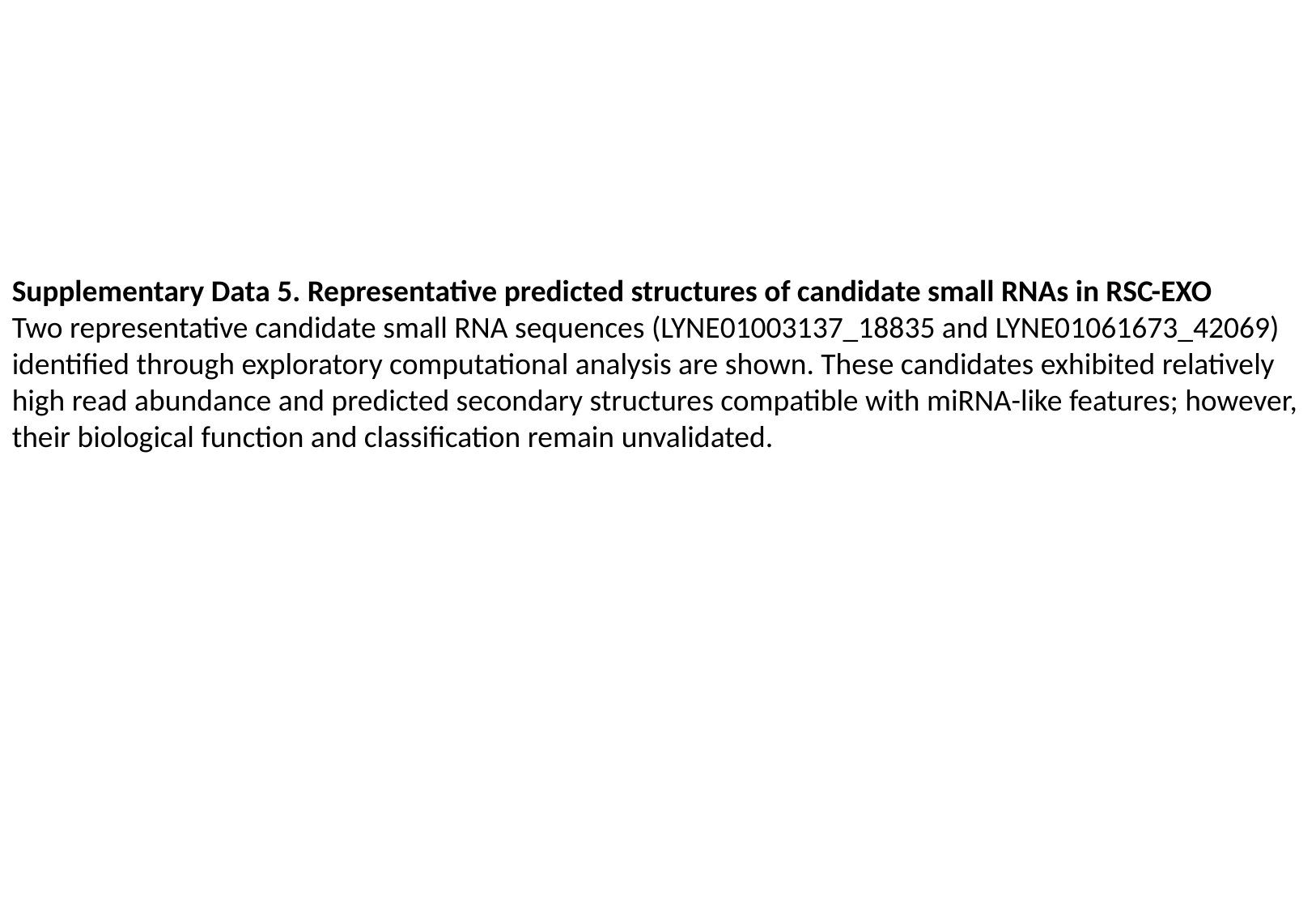

Supplementary Data 5. Representative predicted structures of candidate small RNAs in RSC-EXO
Two representative candidate small RNA sequences (LYNE01003137_18835 and LYNE01061673_42069) identified through exploratory computational analysis are shown. These candidates exhibited relatively high read abundance and predicted secondary structures compatible with miRNA-like features; however, their biological function and classification remain unvalidated.

## Slide 2
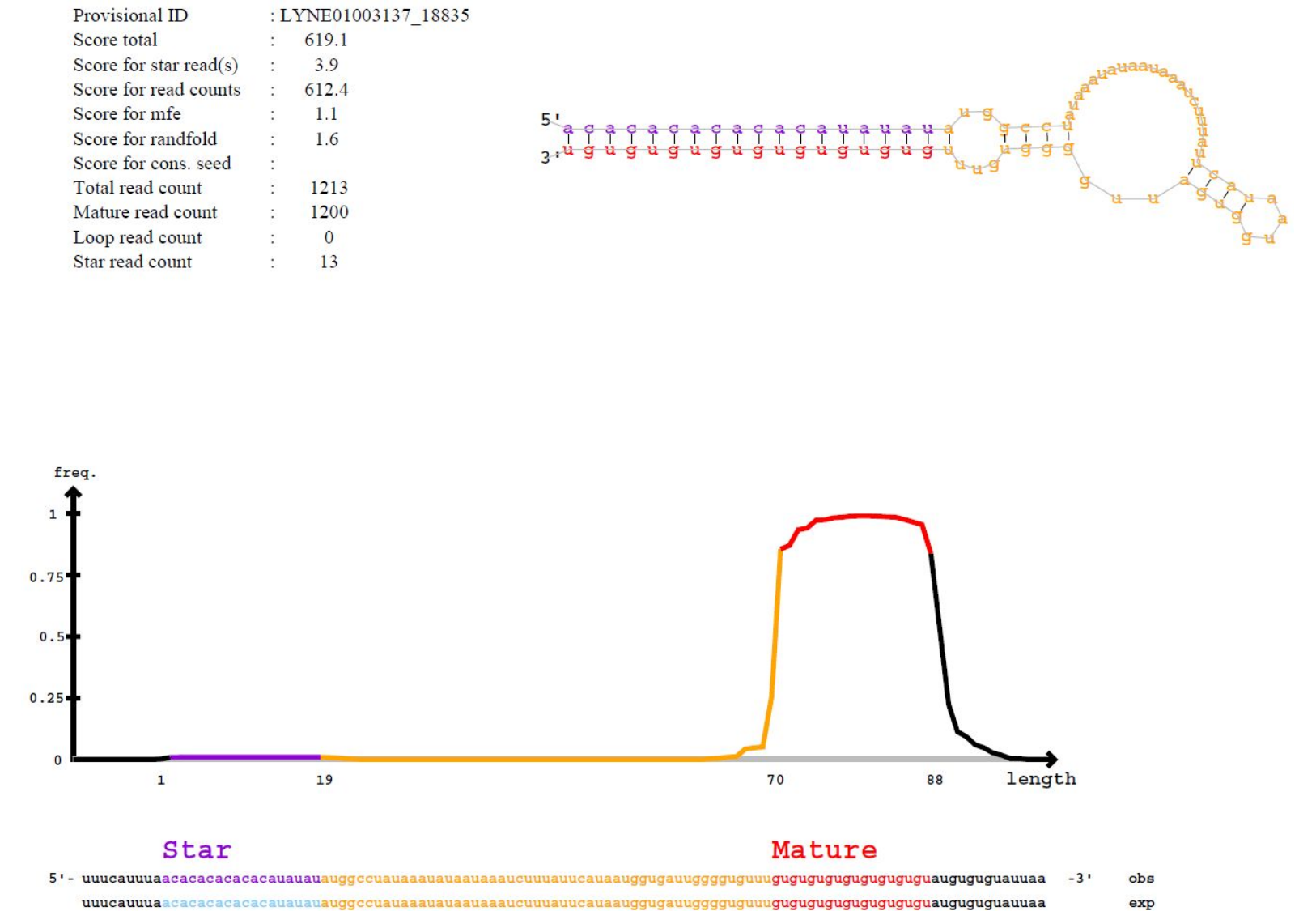

## Slide 3
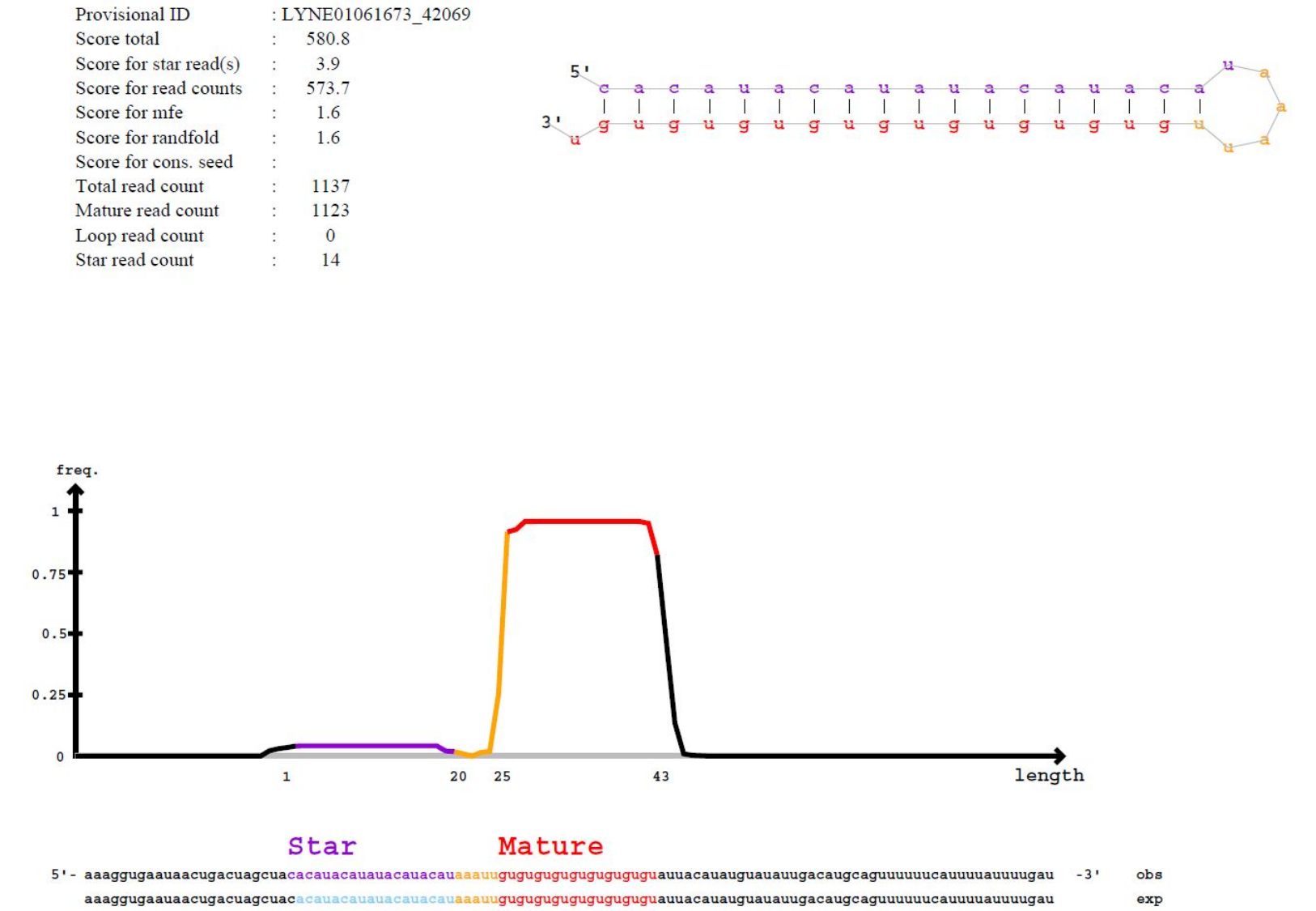

Supplement: Supplementary file 1 [file ijms-27-04938-s001.zip › Supplementary Data 5. Representative predicted structures of candidate small RNAs in RSC-EXO.pptx]
