# Supplementary material for: Characterization of Rosa damascena Callus-Derived Exosome-like Vesicles and Their Multifunctional Activities in Skin-Related Cellular Models
Source: Int J Mol Sci. 2026 May 29;27(11):4938. doi: 10.3390/ijms27114938 (PMC13256896; doi:10.3390/ijms27114938)
Supplement: Supplementary file 1 [file ijms-27-04938-s001.zip › Supplementary Data 6. Uncropped Western blot images.pdf]

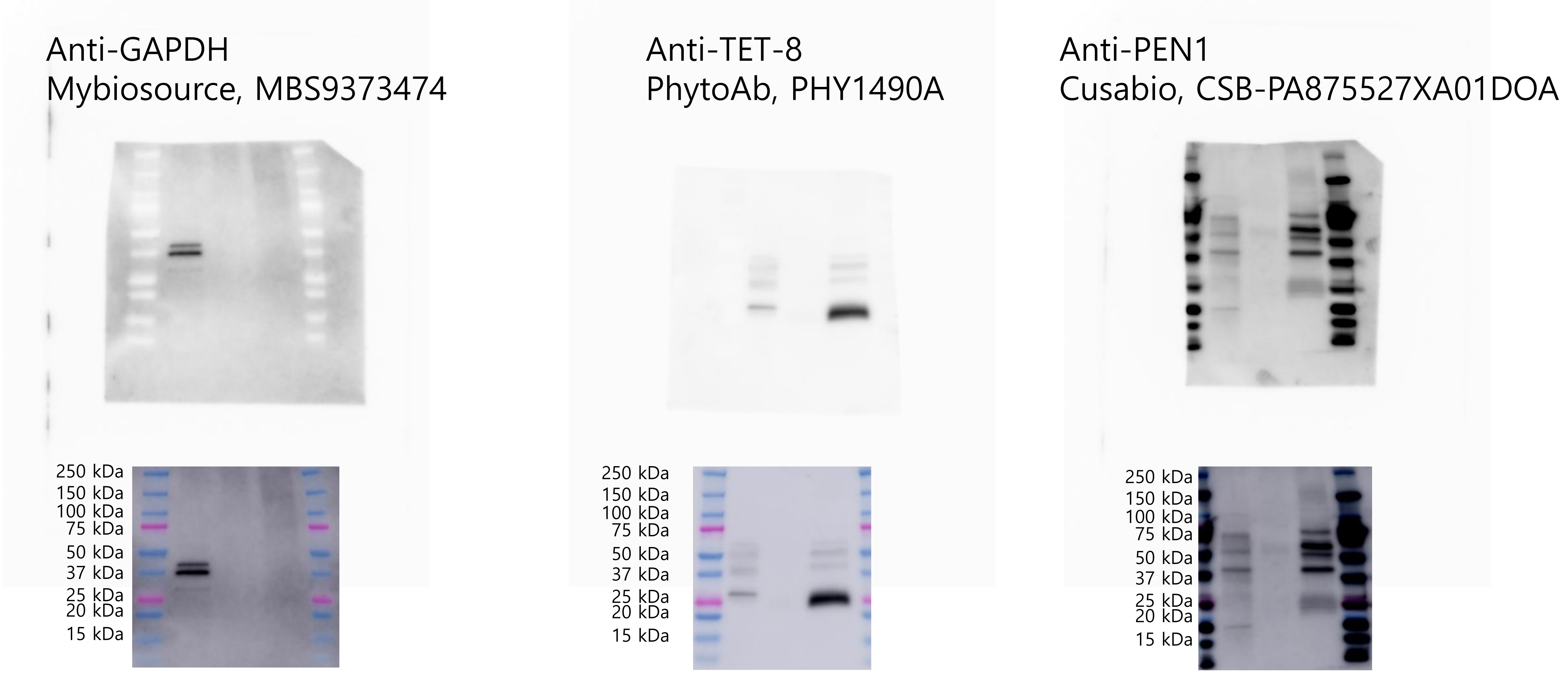

Supplementary Data 6. Uncropped Western blot images.
Uncropped Western blot images corresponding to Figure 1C. The full-length blots are shown with molecular weight markers and lane annotations. These images represent the original, unprocessed data prior to cropping for presentation in the main figures.
